# Technology integration in intensive care medicine through clinical engineering and convergent biotechnology

**DOI:** 10.62838/jccm-2026-0035

**Published:** 2026-07-27

**Authors:** Leonard Azamfirei

**Affiliations:** Department of Anesthesiology and Intensive Care, George Emil Palade University of Medicine, Pharmacy, Science and Technology of Târgu Mureș,Romania

One of the quiet paradoxes of modern intensive care is that as our technology multiplies, our clinical clarity often diminishes. Walk into any contemporary intensive care unit (ICU), and you are confronted with an environment defined by technological abundance: a critically ill patient is surrounded by mechanical ventilators, continuous renal replacement circuits, multi-channel infusion pumps, advanced hemodynamic monitors, and an unrelenting stream of electronic health record notifications. Yet, as every intensivist knows, more technology often translates to more alarms, more fragmented attention, and more distance between the clinician and the bedside. A future ICU defined solely by additional, siloed devices and “black-box” predictive algorithms will not be revolutionary. It will merely be crowded.

The crisis we face is not a lack of tools, but a failure of integration. In medical literature and industry strategy, we often conflate the fields driving these tools, treating bioengineering and biotechnology as interchangeable synonyms. This is a profound conceptual error, and it matters because the way we define these fields dictates how we design tools, train future clinicians, and manage physiology under pressure.

Bioengineering is an indispensable, deeply powerful vertical discipline that applies engineering principles and quantitative modeling to build the extraordinary hardware of modern medicine – the ventilator, the sensor, the extracorporeal circuit [1]. But biotechnology is something entirely different. It is a horizontal, convergent platform that orchestrates these technologies around the complex, dynamic reality of living systems. Classical definitions describe biotechnology as the technological application of biological systems or living organisms to generate knowledge, goods, services, or processes [2,3]. These definitions do not place engineering at the center. They place life at the center.

Even within engineering, further distinctions matter. Medical engineering designs technologies for medicine; clinical engineering ensures that they are selected, implemented, maintained, integrated, and used safely at the bedside [4]. In the ICU, this difference is crucial: invention is not enough. Devices must become inter-operable, clinically usable, and physiologically meaningful. Biotechnology begins where these engineering layers are integrated with biology, data, therapeutics, and clinical reasoning.

These distinctions are decisive.: medical engineering designs technologies for medicine, clinical engineering brings them safely into clinical practice, bioengineering builds technologies for biology., and biotechnology integrates all of them around living systems. The next decade will belong to this convergent biotechnology: digital twins, organoids, organ-on-chip systems, precision medicine, and adaptive clinical decision-support systems. None of these belongs exclusively to one discipline. All emerge from the same deeper shift: biology is becoming technological, and technology is becoming biological [5,6,7,8].

Medicine is the ultimate testing ground for this distinction. Modern care no longer depends on devices, drugs, data, or biological knowledge separately. It depends on their integration. A device without physiological interpretation may be dangerous. A dataset without clinical reasoning may mislead. An algorithm without biological plausibility may produce confidence without wisdom. Medicine is increasingly becoming the place where biology, technology, computation, and judgment must function together.

Nowhere is this more visible than in the ICU.

The ICU is not merely a technologically dense room where engineering tools are deployed. It is physiology under. If we are to survive the oncoming wave of clinical artificial intelligence (AI), digital twins, and autonomous decision-support systems, we must shift our paradigm. The objective cannot merely be technological abundance. The goal must be meaningful biological interpretation at the speed of clinical urgency.

**Fig. 1.**
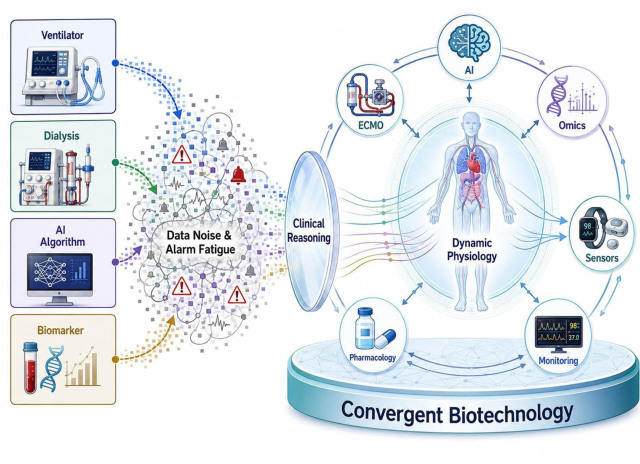
The Critical Care Shift: From Tool-Centric Overload to Patient-Centric Biotechnology Ecosystems. (A) The Bioengineering Silhouette: A vertical paradigm where independent devices and black-box algorithms flood the clinical workspace with fragmented data streams, leading to alarm fatigue and clinical noise. (B) The Biotechnology Platform: A horizontal, integrated convergence platform where advanced computation, predictive models, and mechanical organ support are orchestrated entirely around dynamic host physiology and translated through active clinical reasoning. AI – artificial intelligence; ECMO – extracorporeal membrane oxygenation

An ICU physician is not simply using bioengineering; they are integrating continuous physiological monitoring, laboratory biomarkers, microbiology, imaging, biosensors, mechanical ventilation, extracorporeal support, precision pharmacology, electronic health records, predictive algorithms, computational modeling, and, increasingly, molecular and genomic data. Bioengineering contributes to several of these technologies, but it does not encompass the whole ecosystem. The ICU is therefore not simply a site of technological deployment, but a living laboratory of convergent biotechnology (Figure 1).

A critically ill patient is surrounded by devices, data streams, alarms, predictive models, and complex therapeutic protocols. Yet these technologies are not meaningful simply because they exist. A ventilator is not only a machine; it is an intervention in respiratory mechanics, gas exchange, venous return, pulmonary circulation, acid-base balance, and sometimes survival. A vasopressor is not only a drug; it is a manipulation of vascular tone, cardiac output, microcirculation, tissue perfusion, and cellular oxygen delivery. A biomarker is not only a number; it is a biological signal whose meaning depends on context, timing, trajectory, and physiological interpretation.

This is where biotechnology expands beyond bioengineering. Bioengineering can build the ventilator, the sensor, the extracorporeal circuit, the biosensor, or the monitoring interface. Biotechnology is the larger ecosystem that connects these tools to biology, data, drugs, decisions, and survival. The ICU does not merely use technology. It exposes the biological meaning of technology.

Critical care medicine itself is moving toward this broader frame. Sepsis, acute respiratory distress syndrome, acute kidney injury, shock, and delirium are increasingly understood as heterogeneous biological states rather than uniform entities. Recent work has called for redefining critical illness by focusing less on syndromic labels and more on identifying treatable biological changes [8]. Sepsis guidelines already reflect the complexity of managing dysregulated host response, organ dysfunction, circulatory failure, metabolic disruption, and time-sensitive decisions [9]. The future will not belong to a medicine that names syndromes faster, but to one that identifies treatable biological patterns earlier [9].

That is the promise of medical biotechnology. AI may help detect deterioration before it becomes clinically obvious, and reinforcement-learning approaches have already been explored to personalize sepsis treatment [10]. Yet, many ICU AI applications remain difficult to operationalize clinically [11], bedside implementation requires safety, transparency, and deep clinical integration [12]. Digital twins may transform monitoring and therapeutic titration only if they are dynamically anchored to real patient data, physiological plausibility, and clinical context [13]. Continuous biosensing, omics, closed-loop systems, and organ-on-chip platforms may further reshape therapeutic precision [6,13].

The fundamental danger of the current digital health boom is the temptation to replace physiological reasoning with algorithmic correlation. The ICU does not need more black boxes; it needs physiologically transparent systems. Data without physiology is noise. Technology without biological interpretation is decoration. Algorithms without clinical reasoning are dangerous. A blood pressure value may reflect vasodilation, hypovolemia, myocardial dysfunction, obstruction, dysautonomia, or a drug effect, and, frequently, a combination of several at once. Hypoxemia may be pulmonary, circulatory, hematologic, metabolic, or purely technical. Lactate may signal tissue hypoxia, accelerated glycolysis driven by adrenergic activation, impaired hepatic clearance, or mitochondrial dysfunction during the recovery phase of resuscitation. Numbers do not speak for themselves. Physiology gives them language.

Therefore, the real revolution will not come from replacing clinical physiology with technology, but from embedding physiology into technology. Medical biotechnology must move away from generating isolated instruments, static biomarkers, or opaque algorithms. It must create biologically intelligent systems that preserve the complex, dynamic realities of the human body while helping clinicians act faster, earlier, and more precisely. Physiology is not an archaic preclinical discipline that advanced technology will render obsolete; it is the definitive, common language through which biotechnology becomes medicine. AI predicts physiological deterioration. Ventilators manipulate respiratory mechanics. Vasopressors modify cardiovascular tone. Extracorporeal membrane oxygenation substitutes for failing cardiopulmonary systems. Continuous renal replacement therapy intervenes in metabolic homeostasis. Without physiology, the data stream is deafening. With physiology, biotechnology becomes an extension of the clinician's mind.

This paradigm shift has profound implications for how we train the next generation of critical care specialists. If biotechnology continues to be treated merely as an external branch of engineering, future physicians will be profoundly ill-equipped for the clinical realities already entering our units. Tomorrow's intensivist does not need to become a software engineer, a computer scientist, or a molecular biologist. But they do need to understand how biological signals are generated, captured, processed, modeled, validated, distorted, and ultimately translated into a bedside decision. They require enough physiology to understand the patient, enough data literacy to interrogate the system, enough technological fluency to master the tool, and enough clinical judgment to recognize exactly when the tool is wrong. We must abandon fragmented, disciplinary thinking.

Tomorrow's physician will navigate a dense ecosystem where generative AI, continuous biosensing, computational physiology, digital twins, and closed-loop decision-support systems interact in real time. Training this clinician requires that we stop treating technology as an administrative add-on or a black-box oracle. We must embrace convergent biotechnology as the integrative scientific framework of twenty-first-century medicine.

The intensive care unit shows us the future of medicine because it compresses the entire human and scientific challenge into a single room: life at risk, biology in motion, technology everywhere, decisions under pressure, and physiology as the final common language. In the crucible of the ICU, biotechnology becomes visible not as a product, a device, or a corporate buzzword, but as an active ecosystem through which failing living systems are measured, interpreted, supported, and saved.

Artificial intelligence will be central to this transformation, but only if it is placed in the right conceptual position. It should not become another opaque layer between the clinician and the patient. It should become an interpretive amplifier: a system capable of detecting patterns, anticipating deterioration, integrating complexity, and supporting decisions while remaining accountable to physiology, clinical context, and human judgment. The danger is not that AI will enter the ICU. It already has. The danger is that it will enter as a black box rather than as part of a biologically intelligent ecosystem.

Bioengineering has built extraordinary tools. AI may help those tools perceive, predict, and adapt. But medical biotechnology must now ensure that they speak the language of life.
